# Brief report first report of the in vitro ovicidal activity of camel milk and its fractions on zoonotic-liver fluke (Fasciola gigantica) eggs

**DOI:** 10.1007/s11259-023-10144-8

**Published:** 2023-05-30

**Authors:** Dina A.B. Awad, Amany S. Eldiarby, Mona Abdallah, Ahmed Hamad, Samah M. Abdel Gawad

**Affiliations:** 1 Department of Food Hygiene and Control, Faculty of Veterinary Medicine, Benha University, Moshtohor, 13736 Qalyubia Egypt; 2 Department of Parasitology, Veterinary Teaching Hospital, Faculty of Veterinary Medicine, Benha University, Moshtohor, 13736 Qalyubia Egypt; 3 Department of Zoonoses, Faculty of Veterinary Medicine, Benha University, Moshtohor, 13736 Qalyubia Egypt; 4 Department of Parasitology, Faculty of Veterinary Medicine, Benha University, 13736 Moshtohor, Qalyubia Egypt

**Keywords:** Camel milk, Fascioliasis, Zoonotic parasite, Bioactive proteins

## Abstract

*Fasciola gigantica* is one of the worldwide parasites that cause livestock and human illnesses. Chemotherapy is now the primary therapeutic option for its treatment. Drug abuse has led to the emergence of drug-resistant strains. As a result, there is an urgent need to discover natural and efficient anthelmintics against *Fasciola* spp. The study aims to evaluate the ovicidal activities of camel milk and its fractions on *F. gigantica* eggs. In the in vitro assay of *F. gigantica* eggs were submitted to different concentrations (0.5% and 1%) of camel milk fractions; Camel Milk Whey (CMW), Camel Milk Casein (CMC), and Skimmed Camel Milk (SCM) as well as a positive control (PC) of Nitroxynil (100 mg/ml) and a negative control (NC) with physiological saline. The Egg Hatching Assay (EHA) results showed that camel milk fractions exhibited ovicidal activity, especially CMW, and CMC, which showed 97.58 ± 0.58 and 96.9 ± 1.99 ovicidal activity, respectively, at a concentration of 1% after 15 days of treatment compared to PC, which exhibited 91.75 ± 4.95 ovicidal activity. The egg hatching ratios were 1.67% and 2.33% for CMW and CMC, respectively, compared to 70.17% for the NC and 6% for the PC. The LC_50_ values for CMW and CMC on the 15th day of treatment were 0.20 and 9.13, respectively. From the results above, we can infer that camel milk and its fractions are promising as a new alternative for fascioliasis control.

## Introduction

Zoonotic Liver flukes, including *F. hepatica* and *F. gigantica*, are significant livestock parasites, and infections result in substantial economic losses. *F. hepatica* has a wide geographic distribution, whereas *F. gigantica* occurs in more tropical areas of Africa and Asia (Nukeri et al. 2022). Fascioliasis is of well-known veterinary importance and is an increasing human health problem (Moazeni and Khademolhoseini 2016; Fairweather et al. 2020).

The emergence of anthelmintic resistance is well-known, mainly in gastrointestinal parasites on small-ruminant farms. Reports of resistance to the anti-Fasciola drug and other current drugs are increasing. Similar economic and physiological concerns might exacerbate the development of resistance in *Fasciola* spp., posing a zoonosis hazard (Babják et al. 2021).

This worrying scenario prompts the search for promising anti-Fasciola alternative chemical compounds from natural products for the development of new anthelmintic drugs. In this regard, camel milk appears to be a natural material that can be an alternative because it has been shown experimentally to prevent diseases and disorders such as gastroenteritis, diabetes, and hypertension. (Quan et al. 2008; Sboui et al. 2010).

Camel milk is the most common product in desert regions and contains the same components as bovine milk but exhibits differences in detailed compositions, molecular properties, and processing possibilities (Hailu et al. 2016). Compared to bovine milk, camel milk’s unique health benefits might be linked to differences in its protein composition. Camel milk contains a lot of vitamin C, lactoferrin, lysozyme, lactoperoxidase, minerals (calcium, magnesium, copper, iron, zinc, phosphorus, potassium, and salt), and immunoglobulins, which help fight pathogens and stimulate immunological responses (Mati et al. 2017).

Camel milk has proven to have an antiparasitic effect as it shows anti-schistosomal activity against *Schistosoma mansoni* in infected mice (Maghraby et al. 2005) and in vitro anti-protozoal activity against *Blastocystis* sp. isolated from symptomatic patients (Bakri et al. 2019).

The main goal of this study was to determine the in vitro efficacy of different camel milk fractions as a promising approach for solving the problem of drug resistance in *F. gigantica* compared to the effect of the reference drug (Nitroxynil) used in the field.

## Materials and methods

### Egg collection

The eggs were directly recovered from the bile of each infected buffalo slaughtered in the El-Bassatine abattoir in Cairo, Egypt, by the gallbladder puncture. Each gall bladder was examined individually, evacuated separately in a beaker, mixed with tap water, left to sediment, and decanted supernatant. After several washes with tap water, eggs were suspended in water (500 eggs/ml) and conserved in darkness at 4 °C until used.

### Camel milk Collection

Fresh raw camel milk samples (*Camelus dromedarius*) were collected from Marsa Matruh (31.3543° N, 27.2373° E) and Ras Sedr, South Sinai Governorate (29.5933° N, 32.7178° E), Egypt. All lactating camels were reared in the Egyptian desert by nomads more than two months into lactation, pooled in a sterile stainless-steel container, and immediately refrigerated them. Subsequently, the milk samples were transferred in chilled condition.

### Camel milk fraction preparation

The camel milk fat was skimmed off after centrifugation at 8000 xg for 20 min at 4 °C (SIGMATM 3–18 KS, USA). After fat removal, the remaining skimmed camel milk is referred to as SCM. A portion of SCM was adjusted to pH 4.6 (isoelectric point) (Jenway 3510 pH meter, Cole-Parmer, Staffordshire, UK) with 10% acetic acid and then precipitated by centrifugation at 7871 xg at 4 °C for 20 min. The supernatant fraction (whey proteins), camel milk whey (CMW), and the precipitate (caseins), referred to as camel milk casein (CMC), were dialyzed against phosphate buffer (50 mM, pH 7.8), using 1000 MWCO dialysis bag tubes (Wang et al. 2020). The protein concentration in each fraction was determined according to Bradford (1976).

### In vitro Egg Hatch Assay (EHA)

In the in vitro assay, *F. gigantica* eggs were submitted to different concentrations (0.5% and 1%) of camel milk fractions, CMW, CMC, and SCM, as well as a PC of Nitroxynil 25% (Devomor®, Arabcomed Company, Egypt, 100 mg/ml) and NC with physiological saline. Three replications per group of treatments were involved in this study. Untreated and treated eggs were incubated at 37 °C and examined after 24 and 48 h. under a light microscope to determine the percentage of the egg development (morula stage), then maintained in darkness at 25 °C for 15 days to identify miracidial formation. After this period, the eggs were exposed to daylight for 2 h. Afterwards, 1 ml of 10% (v/v) buffered formalin was added to each tube to stop egg hatching. Eggs were evaluated using an optical microscope (40x magnification). Approximately 50–100 eggs were counted to estimate the proportion of hatched eggs in each tube. Upon observation, eggs were classified into developed eggs (D) and undeveloped eggs (UD). The term developed egg includes the initial stage of embryogenesis, such as incipient morula, and hatched eggs include embryonated eggs containing developed miracidia. The undeveloped eggs have no identifiable miracidium or increase in the number of cells, similar to freshly collected eggs (Solana et al. 2016). The number of developed and undeveloped eggs was counted, the percentage of hatched and developed eggs in each experiment was calculated, and the hatching ratio was calculated according to Hegazi et al. (2018).

### Statistical analysis

Data were analyzed using IBM SPSS statistic version 20, and mean values were used. All analyses were performed in triplicate using one-way ANOVA using descriptive and Duncan. The general linear model determines the effect of the treatment group, time, and their interaction. Finney’s Probit analysis established the LC_50_ and LT_50_ for egg hatch inhibition (Finney 1972). The difference between the means was considered significant at P < 0.05.

## Results

**Fig. 1:**
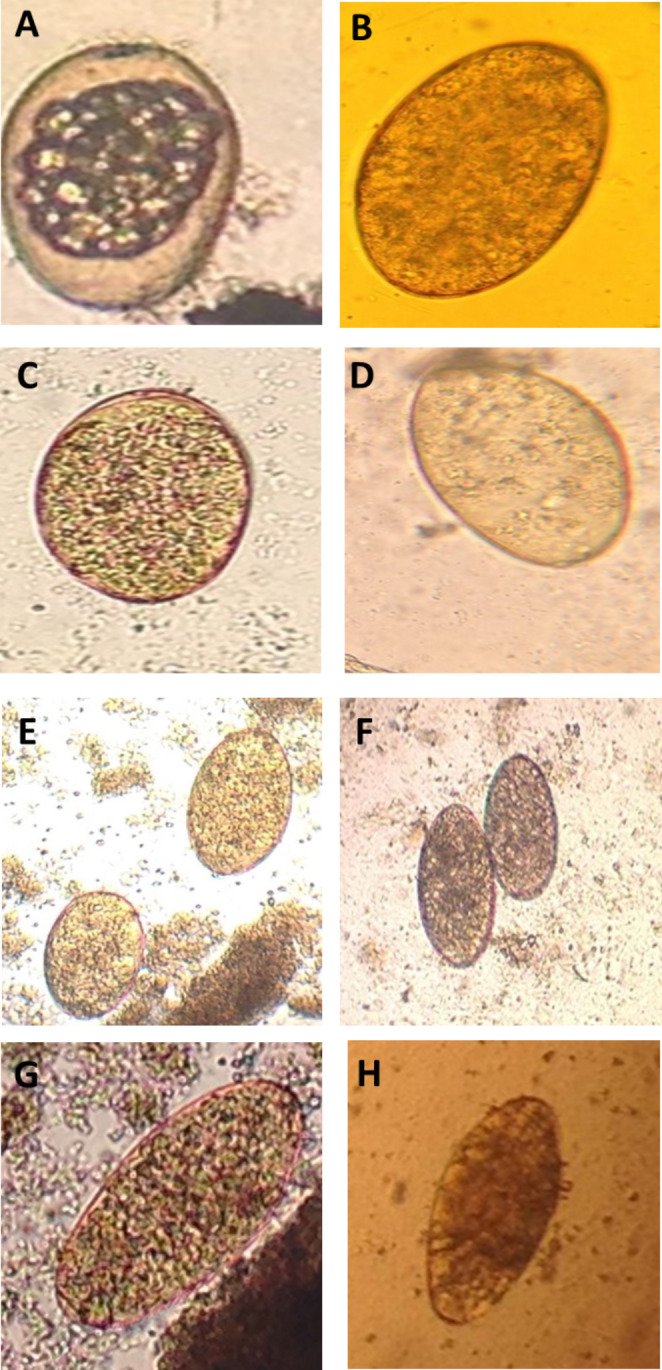
Light microscope of *Fasciola gigantica* eggs treated with different camel milk fractions after 24 h. at 37 °C. (A) physiological saline (negative control); (B) Nitroxynil (100 mg/ml) (positive control); (C) CMW at 0.5%; (D) CMW at 1%; (E) CMC at 0.5%; (F) CMC at 1%; (G) SCM at 0.5%; and (H) SCM at 1%. Magnification: 400x

**Fig. 2:**
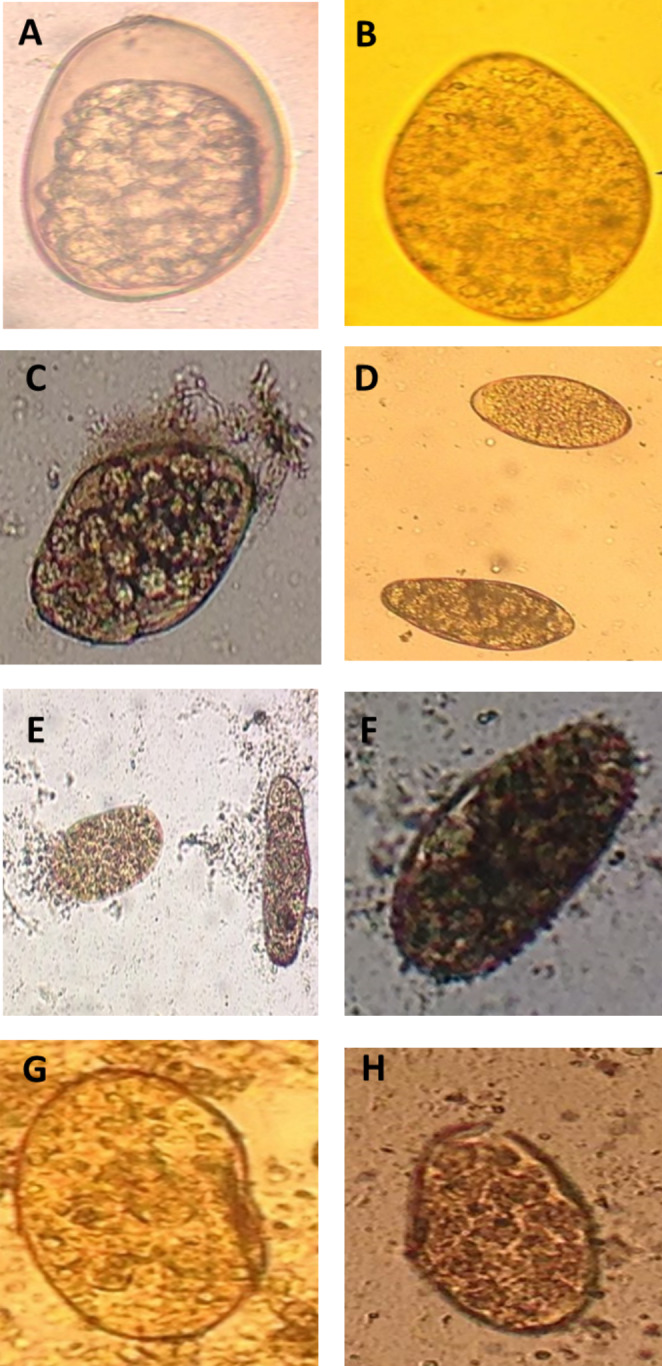
Light microscope of *Fasciola gigantica* eggs treated with different camel milk fractions after 48 h. at 37 °C. (A) physiological saline (negative control); (B) Nitroxynil (100 mg/ml) (positive control); (C) CMW at 0.5%; (D) CMW at 1%; (E) CMC at 0.5%; (F) CMC at 1%; (G) SCM at 0.5%; and (H) SCM at 1%. Magnification: 400x

**Fig. 3:**
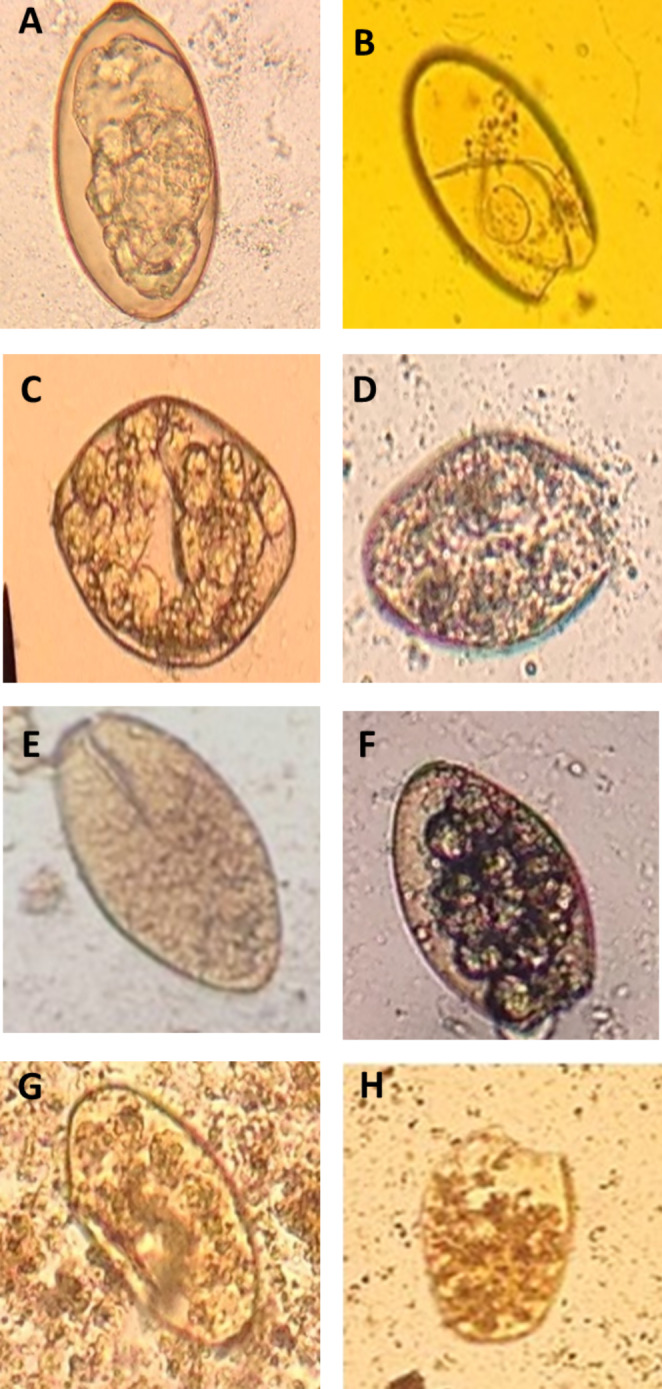
Light microscope of *Fasciola gigantica* eggs treated with different camel milk fractions after 15 days at 37 °C. (A) physiological saline (negative control); (B) Nitroxynil (100 mg/ml) (positive control); (C) CMW at 0.5%; (D) CMW at 1%; (E) CMC at 0.5%; (F) CMC at 1%; (G) SCM at 0.5%; and (H) SCM at 1%. Magnification: 400x

The present study is the first report on the ovicidal activity of camel milk and its fractions against *F. gigantica* eggs isolated from buffalo liver. After 24 and 48 h. of incubation at 37 °C, the cell division stage was observed in both the negative and the treated groups. On the 15th day, the formation of miracidium was observed. After light exposure, the eggs with open operculum were observed, indicating the egress of the miracidium from the egg (Figs. 1, 2 and 3).

The ovicidal effects of the different camel milk fractions with different concentrations on the *F. gigantica* eggs are recorded in Table 1. The result demonstrated that camel milk fractions revealed substantial concentration-dependent ovicidal activity against *F. gigantica* eggs. CMW and CMC at a concentration of 1% exhibited ovicidal activity higher than the SCM and Nitroxynil treated groups. The ovicidal activity of all three camel milk fractions at a concentration of 0.5% was less potent than Nitroxynil and cannot be competed with it.

**Table 1:** Effect of different camel milk fractions at different concentrations and times on the development of *F. gigantica* eggs

Treatment	Concentration	Undeveloped Egg %				Ovicidal activity %	
		24 h	48 h	15 days			
CMW	0.5%	25.67 ± 8.70^ab,2^	31.33 ± 6.48^abcd,2^	89.33 ± 5.45^ab,1^		86.89 ± 6.59^ab^	
	1%	26.83 ± 6.38^ab,3^	56.33 ± 3.75 ^a,2^	98.33 ± 0.42^a^,^1^		97.58 ± 0.58^a^	
CMC	0.5%	17.67 ± 4.05^ab,2^	28.66 ± 2.60^bcd,2^	80.67 ± 2.02^ab,1^		75.96 ± 3.40^bc^	
	1%	24.0 ± 7.88^ab,3^	52.33 ± 15.14^a,2^	97.67 ± 1.49^a,1^		96.9 ± 1.99^a^	
SCM	0.5%	20.67 ± 7.68^ab,2^	29.66 ± 8.41^bcd,2^	73.33 ± 4.05^b,1^		67.3 ± 4.42^c^	
	1%	25.33 ± 7.80^ab,3^	45.67 ± 7.17^ab,2,3^	89 ± 7.25^ab,1^		86.56 ± 8.70^ab^	
Negative control	---	9.0 ± 1.31^b,2^	13.83 ± 4.16^d,1,2^	29.83 ± 6.27^c^,^1^	0.0 ± 0.0^d^		
Nitroxynil 25%	100mg/ml	38.83 ± 4.96^a,2^	47.83 ± 11.89^ab,2^	94.0 ± 3.97^ab,1^	91.75 ± 4.95^ab^		

Note: a, b, and c: No significant difference (P > 0.05) exists between any two means within the same column with the same superscript letter

1, 2, and 3: No significant difference (P > 0.05) between any two means for the same attribute within the row with the same superscript letter

**Fig. 4:**
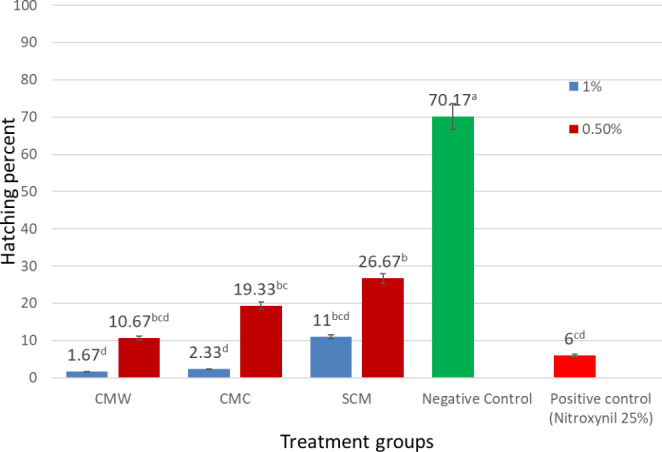
Percentages of hatched eggs after exposure to camel milk fractions with different concentrations than the negative and positive control groups

The percentages of hatching in *F. gigantica* eggs submitted to different camel milk fractions with different concentrations, as well as negative control and Nitroxynil, are shown in Fig. 4. The eggs used in the assay were susceptible to Nitroxynil (100 mg/ml) (PC), with a mean hatching percent of 6.0 ± 3.97%. In the negative control, a hatching percentage of 70.17 ± 6.27% was observed. For camel milk fractions, the results show that the higher the concentration of the camel milk fractions, the lower the percentage of hatched eggs, ranging from 1.67 ± 0.422% to 26.67 ± 4.05%. CMW and CMC, at a concentration of 1%, exhibit the lowest percentage of hatched eggs (1.67 ± 0.422%) and (2.33 ± 1.49%), respectively. The hatching percentages for CMW and CMC at a concentration of 1% were significantly lower than the positive control using the reference drug Nitroxynil (p < 0.05).

**Fig. 5:**
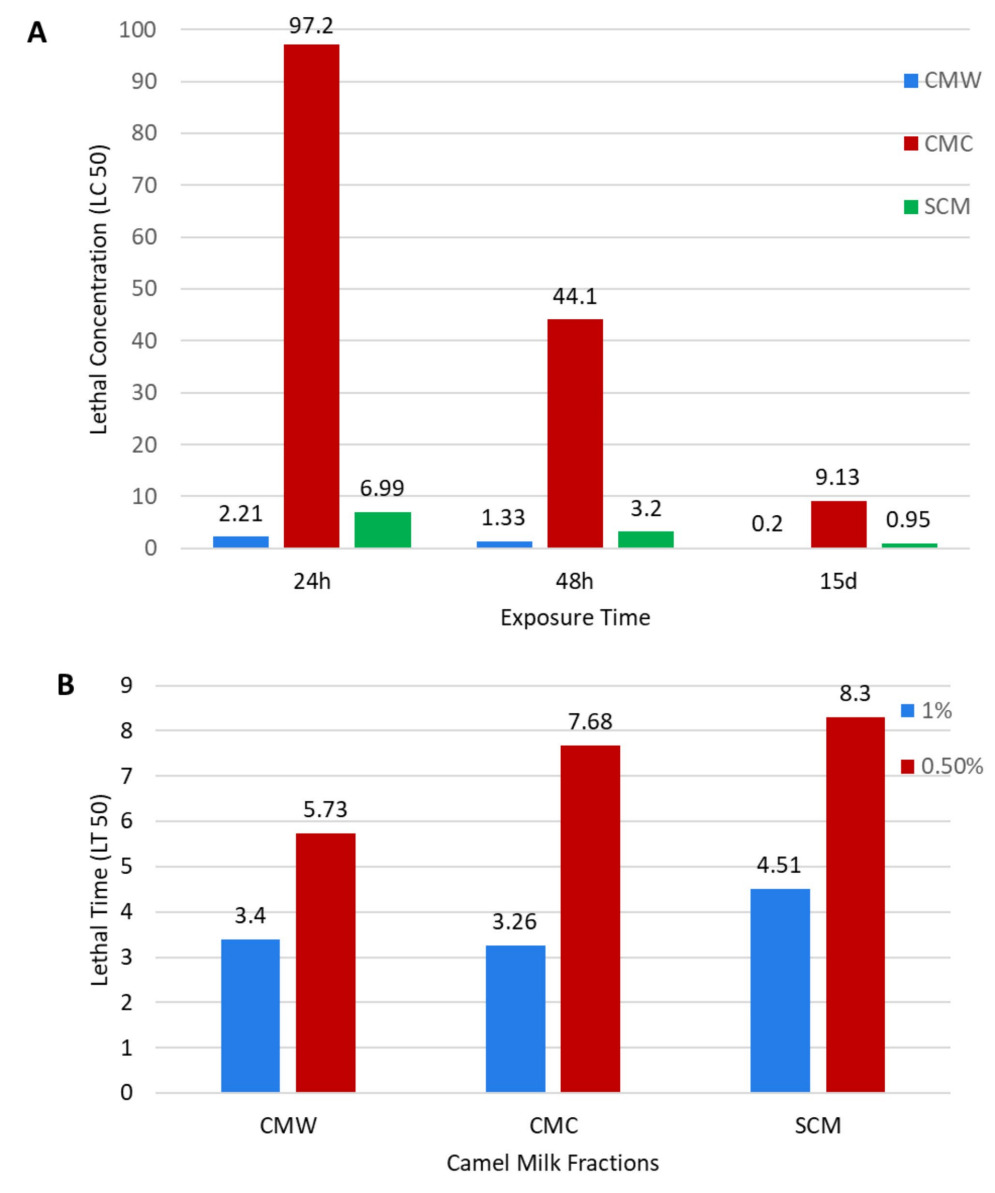
A: Lethal values (LC_50_) of different camel milk fractions at 24 h., 48 h., and 15 days post-exposure on *F. gigantica* eggs, B: Lethal times post-exposure of *F. gigantica* eggs to the different camel milk fractions

The lower the LC_50_, the more lethal the camel milk fraction is to the egg. Generally, the concentration of different fractions required to kill 50% (LC_50_) decreases with exposure time. This indicates that the ovicidal activity of camel milk fractions is time exposure-dependent. The CMW shows the lowest LC_50_ of 0.20 mg/ml in compulsion, as shown by the other milk fractions in Fig. 5A. The lower the LT_50_, the more lethal the camel milk fraction is to the egg. The time required for different camel milk fractions to cause 50% (LT_50_) egg death decreases with an increase in the concentration of the treatments. This indicates that the ovicidal activity of the camel milk fraction is concentration-dependent. The camel milk fraction required the shortest time to cause 50% egg death for all the treatment dosages considered in this study, as shown in Fig. 5B; this confirms that the CMW has higher ovicidal efficacy against *F. gigantica* eggs than the CMC and SCM.

## Discussion

In the present study, we looked for natural sources that have an ovicidal effect against *Fasciola* spp. eggs instead of commercial chemical products. The resistance to anthelmintics commonly used to control fasciolosis is present worldwide (Brennan et al. 2007; Olaechea et al. 2011; Brockwell et al. 2013; Novobilský et al. 2016).

Camel milk has long been recognized in several world regions as a potential cure for ailments such as dropsy, jaundice, TB, asthma, leishmaniasis, or kala-azar (Asresie and Adugna 2014). Camel milk has a potent inhibitory system, and its biological functions may be attributed to the milk’s proteins and peptides (Agamy et al. 1992; FitzGerald and Meisel 2000; Korhonen and Pihlanto-Leppälä 2001).

The current study is, to our knowledge, the first report of the anthelmintic activity of camel milk against *F. gigantica*, reducing egg hatching. Our study revealed that camel milk fractions had an inhibitory effect on egg hatching. Camel milk showed concentration-dependent ovicidal activity at all tested components. Concerning antiparasitic activity against *Fasciola* spp., few reports have evaluated the in vitro effect, making it difficult to compare the results obtained in this study with other scientific reports. In the literature, we found no reports of the efficacy of camel milk fractions against *F. gigantica* eggs. Nevertheless, some studies focus on camel milk’s antiparasitic effect. Maghraby et al. (2005) investigated the anti-schistosomal activity of camel milk on *Schistosoma mansoni*-infected mice. The study suggested that camel milk can be used with anti-schistosomal drugs in schistosomiasis patients. Many other reports have suggested that camel milk exerted an anthelmintic effect against *H. contortus* in sheep (Alimi et al. 2016, 2019), H. *polygyrus* in rodents (Alimi et al. 2018), and protozoa such as *Blastocystis* spp. (Bakri et al. 2019).

The anthelmintic effect of camel milk may be due to the high content of lactoferrin, which acts as a prebiotic and has strong physiological activity in the gastrointestinal tract (Agrawal et al. 2011). Lactoferrin possesses antiparasitic activity against a broad spectrum of species, such as *Pneumocystis carinii* (Cirioni et al. 2000), *Toxoplasma gondii* (Omata et al. 2001), *and Tritrichomonas vaginalis* (Peterson and Alderete 1984; Lehker and Alderete 1992).

*F. hepatica* eggshell is mainly made up of covalently cross-linked proteins. These catecholic proteins are eventually cross-linked or quinone-tanned, sequestering iron, providing exceptional eggshell durability (Ong et al. 1979; Waite and Rice-Ficht 1987). Camel milk, especially whey protein, is rich in lactoferrin, which sequesters iron from the catecholic proteins. Furthermore, quinone-tanned protein material in eggshells is reported to be refractory to proteases, acids, bases, and many organic solvents (Brown 1975). Camel milk is rich in proteolytic enzymes such as lysozyme and lactoperoxidase (Agamy et al. 1992), which may affect these proteins’ integrity. All these factors may be suggested to inhibit Fasciola egg development, which may confirm the high ovicidal effect of CMW over other groups.

The anthelminthic effects of camel milk may also be attributed to its antioxidant activity (Al-Humaid et al. 2009). The antioxidant activity of camel milk is due to the high levels of vitamins B2, C, and E (Abdel Gader and Alhaider 2016) and the high mineral content (Nagy et al. 2013).

Nevertheless, because these are not the only compounds found in camel milk and its fractions, the effects of bioactive compounds cannot be ruled out. Therefore, determining the chemical composition of camel milk that exhibits anthelminthic activity is necessary.

Generally, the LC_50_ of different camel milk fractions decreases with exposure time (Fig. 5A). This indicates that the efficacy of the camel milk fractions in killing *F. gigantica* eggs depends on the time of exposure. This may be due to the absorption of the egg for the bioactive component from the protein fractions, which increases with the time of exposure, increasing the ovicidal activity. The LT_50_ for egg death decreases with an increase in the concentration of the treatments (Fig. 5B). This indicates that the ovicidal activity of the camel milk fractions works in a concentration-dependent manner; this may be attributed to the fact that an increase in the concentration of the components increases the amounts of the bioactive substances to which the egg is exposed, hence increasing ovicidal activity. Unfortunately, there are no such results in the literature using camel milk to which our results can be compared. The present study’s findings confirm the ovicidal activity of fresh camel milk on *Fasciola* eggs, which makes these results promising when searching for effective and non-toxic alternatives.

## Conclusion

One of the significant global challenges in controlling fasciolosis in the veterinary field is the evolution of anthelmintic resistance. Therefore, safe and efficient anthelmintics against *Fasciola* spp. are desperately needed. Our unique outcomes of the current investigation support the idea that camel milk and its fractions offer a new strategy for designing a valuable strategy for controlling liver flukes. For the first time, camel milk whey, followed by camel milk casein, acquired potent ovicidal activity in a concentration-dependent manner against *F. gigantica* eggs, as evidenced by discernible morphological and hatchability differences between the control and camel milk fractions treated groups. The study’s findings offer a promising future for fascinating camel milk proteins to treat this widespread gastrointestinal parasite that affects both people and animals. Further, in vivo studies against different parasite species and stages are required for future rewarding camel milk proteins to control gastrointestinal trematodes parasites.

## Data Availability

Data are available on request.
